# High levels of B-type natriuretic peptide predict weaning failure from mechanical ventilation in adult patients after cardiac surgery

**DOI:** 10.1186/cc10734

**Published:** 2012-03-20

**Authors:** L Hajjar, T Lara, J Almeida, J Fukushima, C Barbas, A Rodrigues, E Nozawa, JL Vincent, F Jatene, J Auler, F Galas

**Affiliations:** 1 Heart Institute, São Paulo, Brazil; 2 Erasme Hospital, Université libre de Bruxelles, Belgium

## Introduction

Failure to wean from mechanical ventilation is related to worse outcomes after cardiac surgery. The aim of the study was to evaluate B-type natriuretic peptide (BNP) as a predictor factor of failure to wean from mechanical ventilation after cardiac surgery.

## Methods

We conducted a prospective and observational cohort study of 101 patients that underwent on-pump coronary artery bypass grafting. BNP was measured postoperatively after ICU admission and at the end of a spontaneous breathing test (SBT). Demographic data, hemodynamic and respiratory parameters, fluid balance, need for vasopressor or inotropic support, lengths of ICU and hospital stay were recorded. Weaning failure was considered as either the inability to sustain spontaneous breathing after 60 minutes or the need for reintubation within 48 hours.

## Results

**Figure 1:**
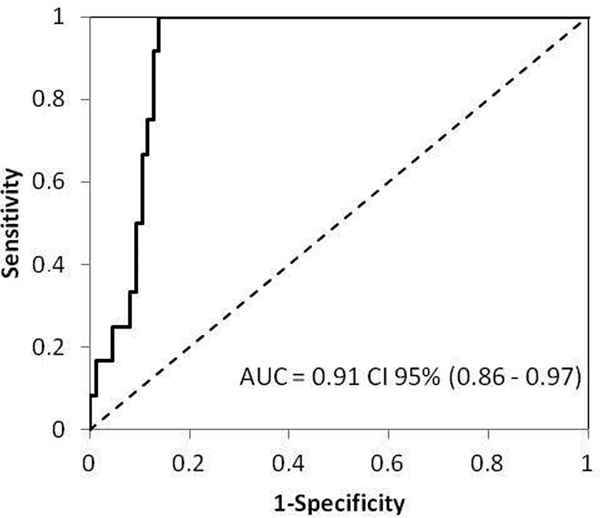
**Area under receiving operating characteristic curve for BNP-2 (at the end of spontaneous breathing test) to predict weaning failure**.

BNP levels were significantly higher both at ICU admission and in the end of breathing test in patients with weaning failure than in the other patients. A BNP concentration of 299 ng/l at the end of the SBT identified weaning failure with 92% sensitivity and 87% specificity, resulting in an area under the curve value of 0.91 (95% CI (0.86 to 0.97), *P *< 0.001) (Figure 1). In a multivariate model, BNP level at the end of SBT was the only predictor of weaning failure from mechanical ventilation.

## Conclusion

BNP was an independent predictor factor of failure to wean from mechanical ventilation after cardiac surgery, which suggests that optimization of the ventricular function must be a goal prior to liberation from mechanical ventilation.

